# Joining a conversation: the problem/gap/hook heuristic

**DOI:** 10.1007/s40037-015-0211-y

**Published:** 2015-09-07

**Authors:** Lorelei Lingard

**Affiliations:** Schulich School of Medicine & Dentistry, Health Sciences Addition, Western University, Rm. 112, N6A 5C1 London, ON Canada

In the writer’s craft section we offer simple tips to improve your writing in one of three areas: Energy, Clarity and Persuasiveness. Each entry focuses on a key writing feature or strategy, illustrates how it commonly goes wrong, teaches the grammatical underpinnings necessary to understand it and offers suggestions to wield it effectively. We encourage readers to share comments on or suggestions for this section on Twitter, using the hashtag: #how’syourwriting?

One of the most powerful shifts a scholarly writer can make has nothing to do with her writing. It has to do with how she thinks about journals. We tend to think that journals exist to publish scholarly manuscripts. But they don’t. They *do* publish scholarly manuscripts, yes, but that’s done in service of a higher purpose: they exist to promote scholarly conversations. The journal-as-conversation metaphor is a powerful conceptual shift because it leads writers to think of their work not as another manuscript, but as the next turn in a conversation.

Imagine yourself joining a conversation at a social event. After you hang about eavesdropping to get the drift of what’s being said (the conversational equivalent of the literature review), you join the conversation with a contribution that signals your shared interest in the topic, your knowledge of what’s already been said, and your intention to add something new that will matter to those participating. When you violate any of those expectations, backs turn or eyes roll, or both.

The conversation metaphor changes our customary notion of what the Introduction of a scholarly paper is meant to accomplish. To position your work as a compelling conversational turn, your Introduction must do three things: (1) Identify a problem in the world that people are talking about, (2) Establish a gap in the current knowledge or thinking about the problem, and (3) Articulate a hook that convinces readers that this gap is of consequence. Ideally, these three elements appear in the first paragraph or two. Consider this example:

Duty hours reform is predicated on the assumption that working fewer consecutive hours will result in more and better-quality sleep hours, which will yield residents who will provide safer patient care. ^1−3^ Existing research is focused primarily on interventions and outcomes related to residents’ on-duty experiences; results of these studies are conflicting and have been used variably to justify or criticize duty hours reform. ^2,4−8^ With very little research into what residents actually do postcall and how they decide what to do, we do not know what influences residents’ decisions about their postcall time. Consequently, it is unclear whether postcall behaviours are particularly entrenched, or what educational or organizational strategies might be implemented to influence them. The lack of such insight is a critical gap in the literature, as researchers have recently found that residents were unlikely to change or improve their sleep habits based solely on an educational intervention to improve their knowledge of sleep physiology principles.^9^ This result begs the ubiquitous knowledge translation question: If improved knowledge is not influential in changing residents’ behaviour, then what would be? [1].

In this example, the problem is that there is conflicting evidence about the impact of duty hours on residents’ practices. The gap is a lack of information about what residents do with their postcall time, and the hook is that this information is necessary to ensure the effectiveness of initiatives designed to improve the situation.

A few distinctions may be helpful as you conceptualize the problem/gap/hook for a paper. First, the problem you’re exploring is not the same as the topic. The following introductory sentence states the topic: *Team communication plays an important role in patient safety.* By comparison, this version states a problem: *Adverse events resulting from error happen at unacceptably high rates in hospital, and ineffective communication among team members is often a contributing factor.* Notice the greater sense of urgency—a problem in the world that matters—conveyed by the second example.

Second, the problem/gap/hook is not the same as your research question and purpose statement. Perhaps you’ve heard that a good Introduction must have a clear question and purpose. These are undoubtedly important features of a research study. They are not, however, the most powerful entrée into a scholarly conversation. A problem/gap/hook approach produces a stronger Introduction. Consider these two examples. The first centres on the question and purpose, while the second uses a problem/gap/hook structure.

Leadership is increasingly recognized as an important competency for physicians. At the same time, collaboration is growing as a value and expectation of health care delivery. What has not been explored is the relationship between leadership and collaboration in physicians’ practice. The purpose of this study was to explore this relationship by asking ‘How do physicians experience leadership and collaboration during their daily team interactions?’

Leadership and collaboration are highly valued and potentially conflicting competencies in medical practice. While there has been attention to leadership and to collaboration individually, little attention has been paid to how they interact. With physicians experiencing increasingly formal expectations that they will lead and collaborate effectively, (e.g., CanMEDS 2015), we require systematic knowledge about how these competencies play out in clinical teams.

Both introductions summarize a gap in knowledge effectively. But the second one, by opening with a problem—these are ‘highly valued and potentially conflicting competencies’—grabs the reader’s attention and inserts itself forcefully into conversations about leadership, collaboration and competency-based education.

Thinking critically about your problem, gap and hook can help you to identify aspects of your Introduction where you may need to be quite strategic. For instance, are you writing about a problem that is truly novel (a rare event), one that is currently being worked on (such as how to implement competency-based assessment), or one that some readers might see as passé or already solved (such as how to design problem-based learning opportunities)? Each of these situations requires a slightly different strategy. Is the gap you have identified one that readers are likely to agree upon, or is there debate about the extent to which more knowledge is required in this area? If readers do not believe that the gap exists, then they may assume your literature review is flawed. If they do not agree that the gap matters—that is, that filling it will change anything—then your story will have no ‘hook’ to draw them in.

The three steps of problem, gap and hook are most useful if you remember that research dissemination is a social and rhetorical act [2]. Journals are static artefacts, but the conversations that they house are dynamic. And journals are increasingly trying to foster the sense of a dynamic conversation: invited commentaries accompany research articles, authors’ blogs are released with online first papers, and author interviews are webcast. The problem/gap/hook heuristic is a powerful way to shape your introduction so that it participates in this scholarly conversation.
